# Graft-versus-host disease after radiation therapy in patients who have undergone allogeneic stem cell transplantation: two case reports

**DOI:** 10.1186/s13256-016-0999-z

**Published:** 2016-07-28

**Authors:** Sarah A. Milgrom, Yago Nieto, Chelsea C. Pinnix, Grace L. Smith, Christine F. Wogan, Gabriela Rondon, L. Jeffrey Medeiros, Partow Kebriaei, Bouthaina S. Dabaja

**Affiliations:** 1 Department of Radiation Oncology, The University of Texas MD Anderson Cancer Center, 1515 Holcombe Boulevard, Houston, Texas USA; 2 Department of Stem Cell Transplantation, The University of Texas MD Anderson Cancer Center, 1515 Holcombe Boulevard, Houston, Texas USA; 3 Department of Pathology, The University of Texas MD Anderson Cancer Center, 1515 Holcombe Boulevard, Houston, Texas USA

**Keywords:** Graft-versus-host disease, GVHD, Radiation therapy, RT, Allogeneic stem cell transplant

## Abstract

**Background:**

Patients who undergo allogeneic stem cell transplantation and subsequent radiation therapy uncommonly develop graft-versus-host disease within the irradiated area. We quantified the incidence of this complication, which is a novel contribution to the field. From 2010 to 2014, 1849 patients underwent allogeneic stem cell transplantation, and 41 (2 %) received radiation therapy afterward. Of these, two patients (5 %) developed graft-versus-host disease within the irradiated tissues during or immediately after radiation therapy.

**Case presentation:**

The first patient is a 37-year-old white man who had Hodgkin lymphoma; he underwent allogeneic stem cell transplantation from a matched unrelated donor and received radiation therapy for an abdominal and pelvic nodal recurrence. After 28.8 Gy, he developed grade 4 gastrointestinal graft-versus-host disease, refractory to tacrolimus and steroids, but responsive to pentostatin and photopheresis. The other patient is a 24-year-old white man who had acute leukemia; he underwent allogeneic stem cell transplantation from a matched related donor and received craniospinal irradiation for a central nervous system relapse. After 24 cobalt Gy equivalent, he developed severe cutaneous graft-versus-host disease, sharply delineated within the radiation therapy field, which was responsive to tacrolimus and methylprednisolone.

**Conclusions:**

We conclude that graft-versus-host disease within irradiated tissues is an uncommon but potentially serious complication that may follow radiation therapy in patients who have undergone allogeneic stem cell transplantation. Clinicians must be aware of this complication and prepared with strategies to mitigate risk. Patients who have undergone allogeneic stem cell transplantation represent a unique population that may offer novel insight into the pathways involved in radiation-related inflammation.

## Background

The management of hematologic malignancies is complex, often involving several types of therapies with toxicities that are heavily influenced by other treatments administered. Allogeneic stem cell transplantation (ASCT) is an important form of treatment for some hematologic cancers. A significant cause of morbidity and mortality after ASCT is graft-versus-host disease (GVHD), an immune response mounted by donor cells against recipient tissues. Several risk factors for GVHD have been identified [1, 2], among them radiation therapy (RT). Our group and others have reported rare cases of GVHD arising within irradiated tissues in patients who have undergone ASCT [3–5]. In this study, we aimed to assess the frequency of this complication.

With the approval of our institutional review board, we retrospectively reviewed records of patients who had undergone ASCT at our institution from 1 January 2010 to 31 December 2014. Those who had received at least one course of RT after ASCT comprised the study population. Disease characteristics, treatment details, and clinical outcomes were retrieved from electronic medical records. GVHD was defined and graded by treating physicians based on clinical and pathologic findings. The RT plans were reviewed to determine the dose that had been delivered to the affected organs.

During the study interval, 1849 patients underwent an ASCT at our institution, 41 (2 %) of whom also received at least one course of RT after ASCT. The baseline characteristics of this cohort are summarized in Table 1. Of these 41 patients, 17 (41 %) experienced acute GVHD (four grade 1, 11 grade 2, and two grade 3); in four cases, the diagnosis of acute GVHD was made after RT (range 21 to 204 days after initiation of RT). In this cohort, nine patients (22 %) developed chronic GVHD (one limited and eight extensive); in four cases, chronic GVHD was diagnosed after treatment with RT (range 22 to 238 days after initiation of RT).

**Table 1:** Patient, disease, and treatment characteristics

Characteristic	Value or number of patients (%)
Age at allogeneic stem cell transplantation, years	
Median (range)	40 (21–69)
Sex	
Male	32 (78 %)
Female	9 (22 %)
Ethnicity	
White	26 (63 %)
Hispanic	10 (24 %)
Black	5 (12 %)
Diagnosis	
Acute myeloid leukemia	9 (22 %)
Acute lymphocytic leukemia	7 (17 %)
Acute biphenotypic leukemia	1 (2 %)
Chronic myeloid leukemia	3 (7 %)
Chronic lymphoid leukemia	1 (2 %)
Mantle cell lymphoma	2 (5 %)
Classical Hodgkin lymphoma	7 (17 %)
Mycosis fungoides	6 (15 %)
Diffuse large B-cell lymphoma	4 (10 %)
Peripheral T-cell lymphoma	1 (2 %)
Donor	
Matched unrelated donor	21 (51 %)
Matched related donor	18 (44 %)
Cord blood	2 (5 %)
Conditioning regimen	
Busulfan/fludarabine	11 (27 %)
Busulfan/clofarabine	5 (12 %)
Busulfan/fludarabine/clofarabine	1 (2 %)
Fludarabine/melphalan	13 (32 %)
Fludarabine/melphalan/rituximab	1 (2 %)
Fludarabine/bendamustine/rituximab	1 (2 %)
Fludarabine/bendamustine/ibritumomab	1 (2 %)
Fludarabine/melphalan/alemtuzumab	2 (5 %)
Melphalan/thiotepa/fludarabine/cyclophosphamide	1 (2 %)
Busulfan/clofarabine/gemcitabine	4 (10 %)
Fludarabine/cyclophosphamide/2 Gy TBI	1 (2 %)
Time from ASCT to RT, days	
Median (range)	299 (45–1715)
RT dose, Gy	
Median (range)	23.4 (2–44)
Number of RT fractions	
Median (range)	12 (1–22)

*ASCT* allogeneic stem cell transplantation, *RT* radiation therapy, *TBI* total body irradiation

In two patients (5 % of the cohort), GVHD developed during or immediately after RT within the irradiated tissues. In a third case, hemorrhagic esophagitis developed 7 days after completion of craniospinal irradiation (CSI). Although an esophageal biopsy was morphologically suggestive of GVHD, the clinical presentation was not consistent with this diagnosis, and the symptoms resolved with steroids alone. Therefore, the symptoms were attributed to RT-induced inflammation, and this case was not included. The two cases of GVHD following RT are described below.

## Case presentation

### Case 1

An otherwise healthy 37-year-old white man had chemorefractory classic Hodgkin lymphoma, treated with the following regimens: (1) doxorubicin, bleomycin, vinblastine, and dacarbazine; (2) ifosfamide, carboplatin, and etoposide; (3) brentuximab; (4) gemcitabine and vinorelbine; (5) bendamustine; (6) everolimus; (7) sirolimus and vorinostat; (8) lenalidomide; and (9) dexamethasone, cytarabine, and cisplatin. He then underwent an ASCT from a 10/10 matched unrelated donor, with a fludarabine and melphalan conditioning regimen.

**Fig. 1:**
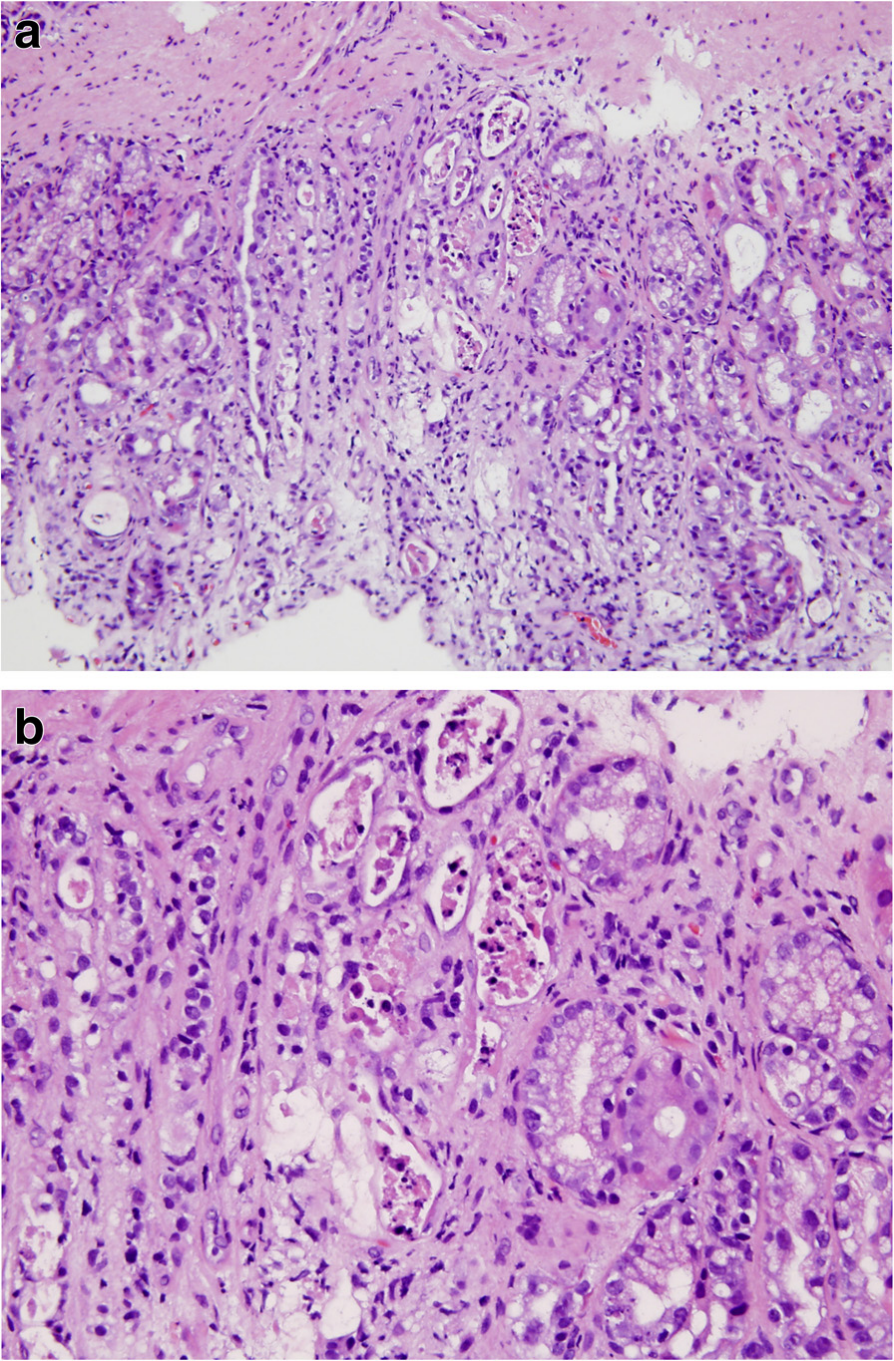
**a** Full thickness of gastric antrum showing denuded epithelium, loss of glands, dilated glands, and relatively few inflammatory cells in the lamina propria. **b** High magnification showing dilated gastric glands with eosinophilic granular debris and apoptotic nuclear fragments. These findings support grade 4 graft-versus-host disease

Three months after the ASCT, the disease relapsed in his abdominal and pelvic lymph nodes. He was treated with intensity-modulated RT to his para-aortic and pelvic lymph nodes, starting on day 119 after the ASCT. At that time, no evidence of GVHD was present, and the tacrolimus was tapered off. The plan was to treat his abdominal and pelvic lymph nodes with a total dose of 39.6 Gy in 22 fractions. However, after completing 28.8 Gy in 16 fractions, he experienced severe abdominal pain, nausea, vomiting, and copious watery diarrhea. An endoscopy revealed an erythematous gastrointestinal mucosa with superficial ulcers. Biopsies taken throughout his gastrointestinal tract were consistent with GVHD. In his stomach and duodenum, the mucosa showed loss of glands, dilated glands with eosinophilic and granular debris, and increased apoptotic cells (Fig. 1). In his colon, increased apoptotic cells and loss of glands were identified.

**Fig. 2:**
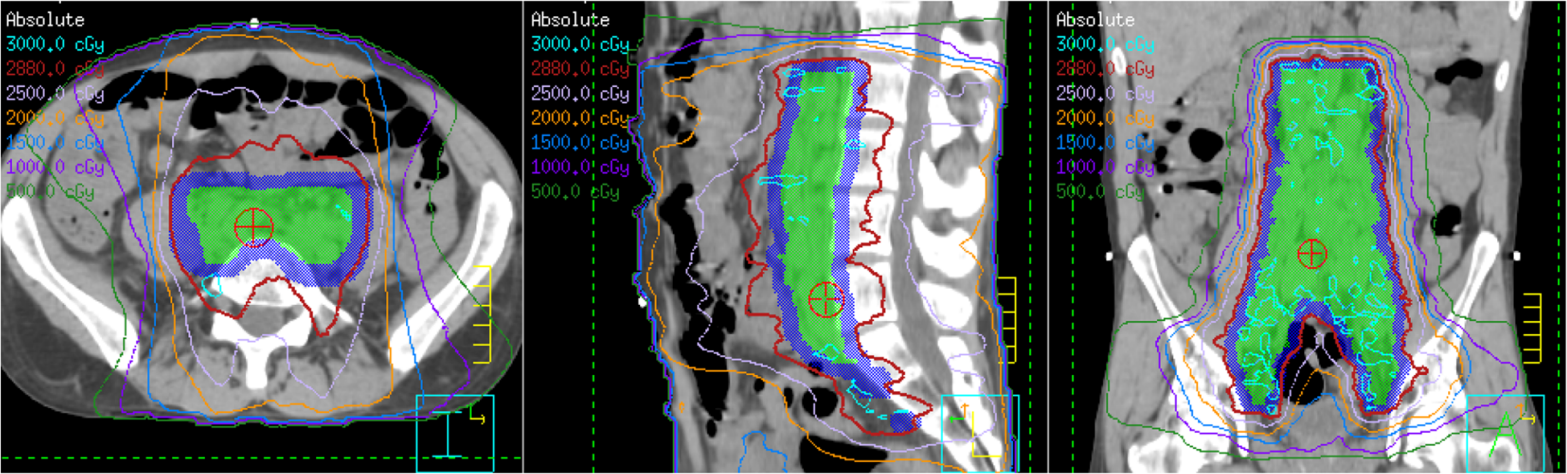
Intensity-modulated radiation therapy plan for Case 1 shows the 28.8 Gy that had been delivered when graft-versus-host symptoms appeared. *Green color wash* indicates clinical target volume; *blue color wash* indicates planning target volume

These findings led to a diagnosis of grade 4 GVHD of the gastrointestinal tract, experienced after 28.8 Gy. His bowel, contoured as “bowel space,” received a maximum dose of 31.8 Gy and mean dose of 14.8 Gy. The volume of bowel space that received ≥30 Gy was 39 cc, ≥20 Gy was 1400 cc, and ≥10 Gy was 2450 cc. His stomach received a maximum dose of 25 Gy and mean dose of 2.9 Gy. The volume of stomach that received ≥20 Gy was 4.3 cc and ≥10 Gy was 31 cc (Fig. 2). Initial treatment with tacrolimus and steroids (2 mg/kg/day) had no effect, but subsequent pentostatin and photopheresis produced a good response. At last follow-up, 6 months after RT had been stopped, his GVHD was quiescent on photopheresis and tacrolimus, with no evidence of active Hodgkin lymphoma in his abdomen or pelvis.

### Case 2

As previously reported [4], an otherwise healthy 24-year-old white man with relapsed Philadelphia-positive B-cell acute lymphocytic leukemia was treated initially with cyclophosphamide, vincristine, doxorubicin, dexamethasone, cytarabine, and methotrexate (hyper-CVAD) with dasatinib. Disease relapse in his central nervous system (CNS) and bone marrow during maintenance therapy was salvaged with augmented hyper-CVAD and dasatinib, followed by a 10/10 human leukocyte antigen (HLA)-matched related ASCT from his sister, with a busulfan and clofarabine conditioning regimen.

On day 82 after the ASCT, he presented with a headache; he was diagnosed as having an isolated CNS relapse and was treated with rituximab, asparaginase, dasatinib, high-dose methotrexate, and intrathecal cytarabine, followed by consolidative CSI. The CSI was with proton therapy, to a total dose of 24 cobalt Gy equivalent (CGE) in 12 fractions. His brain was treated with right and left posterior oblique beams, and his spine was treated with three posterior-anterior beams. The CSI was begun on day 197 after the ASCT, and no evidence of GVHD was present at that time. The tacrolimus dose was reduced during RT.

One month after completing CSI, he developed severe dermatitis within the RT portals and conjunctivitis, keratopathy, and conjunctival ulceration. The dose delivered to his skin had been 22 CGE [4]. A skin biopsy showed inflammatory cell-poor interface dermatitis with vacuolar alterations of the basal keratinocytes and dyskeratotic cells, consistent with grade 2 to 3 GVHD. Treatment with tacrolimus and methylprednisolone (2 mg/kg/day) resulted in resolution of his cutaneous GVHD; however, keratoconjunctivitis sicca persisted despite prednisolone ophthalmic drops. His cutaneous GVHD returned several months later, both within and outside the RT field. This extensive chronic GVHD progressed despite steroids, tacrolimus, and photopheresis, manifesting as ulcerations, scleroderma-like changes, and chronic osteomyelitis that necessitated bilateral above-the-knee amputations. He died of aspiration pneumonia and respiratory failure 4.5 years after the ASCT, with no evidence of leukemia.

## Conclusions

GVHD is a potentially serious complication that may follow RT in patients who have undergone ASCT. Cases have been reported [4–6]; however, the incidence of this complication was unknown previously. We identified 41 sequential patients who received RT after ASCT, two (5 %) of whom developed clinically significant GVHD within the irradiated tissues during or immediately after RT, despite delivery of relatively low doses to the affected organs. Radiation-induced inflammation was an alternative diagnosis that was considered; however, both the clinical and pathological findings were consistent with GVHD. We conclude that GVHD following RT is uncommon; however, this diagnosis should be considered in patients who have undergone ASCT and who develop RT-related side effects that are more severe than expected.

## Discussion

Given the study design, conclusive demonstration of a causal relationship between RT and GVHD is not possible. However, GVHD developed within the irradiated tissues during or immediately following RT, in patients who were without evidence of GVHD previously. Furthermore, in Case 2, cutaneous GVHD was strictly demarcated within the irradiated area [4]. These findings strongly suggest a causal relationship.

The small number of events in this study precludes the identification of factors that might modify the risk of GVHD. It is notable, however, that the doses of immunosuppressive agents were tapered around the time of RT in both cases. This observation suggests that immunosuppressive therapy should not be reduced during this period, even if patients are free of clinically apparent GVHD at the time of RT.

Theoretically, RT can trigger GVHD via local cellular damage and induction of pro-inflammatory pathways. Multiple inflammatory mediators are activated, upregulated, or released in response to ionizing radiation, such as NF-kB, TNF-α, TGF-β, GM-CSF, COX-2, ICAM-1, IL-1, IL-6, IL-8, IFN, c-Fos, c-Myc, c-Jun, and extracellular nucleotides [6–11]. The resulting chemotactic signals lead to rapid recruitment of diverse leukocyte subgroups into the irradiated area [12–14]. RT also upregulates expression of major histocompatibility complex (MHC) class I/II antigens by cancer cells, rendering them more sensitive to T cell recognition through antigen presentation by dendritic cells [13, 15, 16]. RT may well have similar effects on normal tissues. For patients who have had an ASCT, this immune stimulation may cause dendritic cells to present host antigens to donor T cells, predisposing to the development of GVHD.

The immune response induced by ionizing radiation is an area of fervent study, both in the laboratory and in the clinic. Patients who have undergone ASCT represent a unique population that may offer additional insight into the pathways involved in radiation-related inflammation. Furthermore, clearer understanding of the mechanisms by which RT induces GVHD may enable the development of therapeutic interventions. RT is a highly effective form of treatment for hematologic malignancies, but clinicians must be able to recognize risk factors for toxicity, including GVHD, and strive to develop strategies to mitigate morbidity.

## Abbreviations

ASCT, allogeneic stem cell transplantation; CGE, cobalt Gray equivalent; CNS, central nervous system; CSI, craniospinal irradiation; GVHD, graft-versus-host disease; HLA, human leukocyte antigen; hyper-CVAD, cyclophosphamide, vincristine, doxorubicin, dexamethasone, cytarabine, and methotrexate; MHC, major histocompatibility complex; RT, radiation therapy
